# Functional membrane androgen receptors in colon tumors trigger pro-apoptotic responses *in vitro *and reduce drastically tumor incidence in *vivo*

**DOI:** 10.1186/1476-4598-8-114

**Published:** 2009-12-01

**Authors:** Shuchen Gu, Natalia Papadopoulou, Eva-Maria Gehring, Omaima Nasir, Konstantinos Dimas, Shefalee K Bhavsar, Michael Föller, Konstantinos Alevizopoulos, Florian Lang, Christos Stournaras

**Affiliations:** 1 Department of Physiology, University of Tübingen, Germany; 2 Department of Biochemistry, University of Crete, Medical School, Heraklion, Greece; 3 Center of Basic Research, Biomedical Research Foundation Academy of Athens, Athens, Greece; 4 Medexis-Biotech SA, Kryoneri Athens, Greece

## Abstract

**Background:**

Membrane androgen receptors (mAR) have been implicated in the regulation of cell growth, motility and apoptosis in prostate and breast cancer. Here we analyzed mAR expression and function in colon cancer.

**Results:**

Using fluorescent mAR ligands we showed specific membrane staining in colon cell lines and mouse xenograft tumor tissues, while membrane staining was undetectable in healthy mouse colon tissues and non-transformed intestinal cells. Saturation/displacement assays revealed time- and concentration-dependent specific binding for testosterone with a K_D _of 2.9 nM. Stimulation of colon mAR by testosterone albumin conjugates induced rapid cytoskeleton reorganization and apoptotic responses, even in the presence of anti-androgens. The actin cytoskeleton drug cytochalasin B effectively inhibited the pro-apoptotic responses and caspase-3 activation. Interestingly, *in vivo *studies revealed that mAR activation resulted in a 65% reduction of tumor incidence in chemically induced Balb/c mice colon tumors.

**Conclusion:**

Our results demonstrate for the first time that functional mARs are predominantly expressed in colon tumors and that their activation results in induction of anti-tumor responses *in vitro *and extensive reduction of tumor incidence *in vivo*.

## Introduction

Scientific evidence accumulated in recent years points to the existence of membrane androgen receptors (mAR), triggering rapid, non-genomic signals. Although the exact molecular identity of mAR still remains unknown, non-genomic androgen actions manifested within minutes have been reported in various cell types including macrophages and T cells [1,2], LNCaP [3,4], T47D [5], MCF7 [6], DU145 [7-9], C6 [10], PC12 [11] or VSMC cells [12]. These effects are clearly different from those manifested upon activation of the intracellular androgen receptors (iAR) mediating genomic androgen signals resulting in receptor dimerization, nuclear translocation and subsequent activation of androgen-specific target genes (reviewed in [13]).

The mAR-dependent signaling was recently characterized in detail in prostate and breast cancer cell lines (reviewed in [14-17]). Using non-permeable androgen derivatives that do not bind to iAR, it was shown that mAR activation resulted in actin reorganization regulated by mechanisms involving small GTPases [8,18]. Furthermore, it was shown that mAR activation induced profound apoptotic regression of prostate cancer cells *in vitro *and in mouse xenografts *in vivo *[7,19] and suppressed cell growth and motility [6,19]. Finally, most recent studies have implicated key pro-survival and pro-apoptotic gene products such as AKT, NF-κB, Bad, Fas and caspase-3 in the regulation of the apoptotic response induced by mAR activation in prostate cancer cells [9].

Taken together, these studies clearly established that functional mARs trigger strong anti-tumorigenic effects, implying a potential role of mAR as a novel target for the development of selective cancer treatments (reviewed in [17]). However, it remained elusive whether mARs are also expressed in other tumors and whether their activation could result in the induction of anti-tumorigenic effects similar to the ones described in prostate and breast cancer cells. Colon tissues are known to express functional nuclear hormone receptors (reviewed in [20]), and specific AR, ERα and ERβ genotypes have been associated with colon cancer [21]. Moreover, administration of androgens has been linked to the promotion of colon cancer tumorigenesis in rats [22]. On the other hand, steroid hormones induced tumor growth remission in xenotransplanted adenocarcinomas in nude mice [23], arguing for a more complex role of steroid hormones in colon cancer. Since the membrane androgen receptor, in contrast to the classical intracellular androgen receptor, induces tumor regression in target tissues (reviewed in [17]), we sought to determine the expression and functional status of mAR in colon cancer. To this end, we used colon cancer tissues isolated from mice xenograft tumors and from two established colon cancer cell lines (Caco2 and HCT116 cells). As a result, testosterone binding sites were expressed in the membrane of colon cancer cells and qualify as bona fide membrane androgen receptors as assessed by radioligand binding studies, Scatchard analysis and displacement assays. The activation of those receptors with non-permeable testosterone derivatives triggered rapid and profound actin and tubulin cytoskeleton reorganization and induced pro-apoptotic responses. Finally, treatment of Balb/c mice with testosterone albumin conjugates resulted in considerable anti-tumor activity *in vivo*. Our findings provide strong evidence for the anti-tumorigenic activity of functional mAR in colon cancer both *in vitro *and *in vivo *and further support previous reports on the clinical significance of mAR targeting for cancer treatment.

## Materials and methods

### Cell cultures

The Caco2 and HCT116 human colon cancer cell lines and the non transformed intestinal IEC06 cells were obtained from the American Type Culture Collection (Manassas, VA) and were studied between passages 55 and 70.

### Preparation of steroid solution

Before each experiment testosterone-3-(O-carboxymethyl)oxime human serum albumin, (testosterone-HSA or Testo-HSA; Sigma) was dissolved in serum-free culture medium at a final concentration of 10^-5 ^M. This stock solution was incubated for 30 min at room temperature with 0.3% charcoal and 0.03% dextran, centrifuged at 3,000 × g and passed through a 0.45 μm filter to remove any potential contamination with free steroid. This is highly important for the interpretation of the results to disconnect any possible intracellular testosterone- and/or iAR-interference with the effects mainly induced by the mAR activation. Testosterone HSA, estradiol and dihydrotestosterone (DHT) (Sigma) solutions were used at a final concentration of 10^-7 ^M throughout the study unless otherwise mentioned. All treatments and incubations with steroids including apoptosis assays were performed in serum-containing medium. Testosterone-HSA-FITC or control HSA-FITC constructs were generated by conjugating Testosterone-HSA or HSA with FITC (Sigma) using standard techniques.

### Preparation of paraffin blocks from HCT116 colon cancer xenografts

HCT116 cells or p53-deficient HCT116 (HCT116p53-/- cells, kindly donated by Dr. Vogelstein) were injected subcutaneously at the axillary region of 7-9 weeks old male SCID (NOD.CB17 Prkdcscid) mice (Jackson Laboratories/Charles River Laboratories, L'Arbresle, France) according to the British practice of bilateral trocar implants as described previously [24]. Each inoculum contained 10^6 ^cells exponentially growing at the time of harvesting. When tumors reached about 1000 mm^3 ^in size, animals were sacrificed and tumors were excised and fixed in buffered formalin (4%) embedded in paraffin. 4-μm sections were prepared with the aid of a Leica microtome (model RM2125). Subsequently, sections were stained with hematoxylin-eosin and examined under a microscope to assess the histological phenotype of the tumor, the type and degree of differentiation, and the presence of regressive changes. Other sections were de-paraffinized and subjected to standard immunofluorescence analysis using fluorescent Testo-HSA-FITC or HSA-FITC control conjugates as described in the following section. All animals were treated according to the Greek law and the instructions of the European council (86/609 and ETS123, respectively) governing the use and handling of animals in experiments.

### Immunofluorescence analysis and confocal laser scanning microscopy

Cells were cultured on glass cover slips with testosterone-HSA-FITC or control HSA-FITC using the concentrations and the incubation periods indicated in the figure legends. For testosterone-HSA-FITC staining, cells or specimens were washed twice with PBS containing 1.5% FBS for 1.5 min and incubated for 1 h with 1% BSA in PBS at room temperature. After two washes with PBS/1.5% FBS cells were exposed to 10^-7 ^M testosterone-HSA-FITC, while control cells were incubated with 4 × 10^-7 ^M HSA-FITC for 1 h at room temperature. Nuclei were stained with DRAQ5™ (Biostatus Limited) or TO-PRO-3 (Invitrogen). After two washes with PBS/1.5% FBS and fixation with 0.5% paraformaldehyde for 30 min cells were washed twice with PBS/1.5% FBS for 3 min and mounted with slow anti-fade. For direct fluorescence microscopy of F-actin, cells were fixed with 3% paraformaldehyde in PBS for 30 min, permeabilized with 0.5% Triton X-100 in PBS (10 min) and incubated with rhodamine-phalloidin (Molecular Probes, Eugene, OR, 1:100 dilution) for 40 min in the dark. For indirect immunofluorescence staining cells were incubated for 2 h at room temperature with mouse monoclonal anti-tubulin (Cell signaling, 1: 1000 dilution). Secondary FITC-conjugated rabbit anti-mouse IgG (Invitrogen) was used at a 1:200 dilution. Nuclei were stained with DRAQ5™ (Biostatus Limited). Slides were mounted using the ProLang^® ^Gold Antifade reagent (Invitrogen). All specimens were examined with a BH-2 microscope (Olympus Corp., Lake Success, NY) equipped with epifluorescence illumination. Confocal microscopy was performed on a Zeiss LSM 5 EXCITER confocal laser-scanning module (Carl Zeiss, Göttingen, Germany) and images were analyzed with the software of the instrument.

### Binding assays

For membrane preparation Caco2 cells cultured in five 150 cm^2 ^flasks without serum, were washed twice with PBS, removed by scraping, and centrifuged at 1,500 g for 5 min. Pelleted cells were homogenized by sonication in 50 mM Tris-HCl buffer, pH 7.4, containing freshly added protease inhibitors (10 μg/ml PMSF and Roche complete protease inhibitor tablets). Unbroken cells were removed by centrifugation at 2,500 g for 15 min. Membranes were obtained by centrifugation at 45,000 g for 1 h and washed once with the same buffer. Protein concentration was measured by the method of Bradford using reagents from Bio-Rad (Hercules, CA).

### Binding conditions

Saturation binding experiments were performed in a final volume of 0.1 ml containing cell membranes at a final protein concentration of 1.2 mg/ml and at least seven concentrations of [^3^H] testosterone (Amersham, GE Healthcare, United Kingdom) ranging from 2 to 100 nM. For displacement binding experiments, cell membrane preparations at a final concentration of 1.2 mg/ml were incubated with 5 nM [^3^H] testosterone in the absence or in the presence of different concentrations of unlabeled steroid (DHT, estradiol), ranging from 10^-12 ^to 10^-6 ^M. Non-specific binding was estimated in the presence of 5 μM DHT. In both types of binding experiments, after an overnight incubation at 4°C, bound radioactivity was separated by filtration under reduced pressure through GF/A filters previously soaked in 0.5% polyethylenimine (PEI) in water and rinsed three times with ice-cold Tris-HCl buffer. Filters were mixed with 10 ml scintillation cocktail (03999, Fluka), and bound radioactivity was counted in a scintillation counter (1415 Liquid Scintillation Counter, Wallac) with 60% efficiency for Tritium. The *K*_D _and B_max _values for the membrane binding sites were determined from Scatchard plots based on saturation bindings.

### Cell-surface biotinylation and Western blotting

Caco2 cells were washed twice with ice cold PBS and surface biotinylated with 0,5 mg/ml sulfo-NHS-SS-biotin (Pierce, Rockford, IL) for 30 min at 4°C. Cells were subsequently washed twice with PBS and lysed for 1 hour in RIPA buffer (10 mM Tris, pH 7.4, 150 mM NaCl, 1.0 mM EDTA, 0.1% SDS, 1.0% Triton X-100, 1.0% sodium deoxycholate) with protease inhibitors (Roche, Mannheim, Germany). Then, 500 μg protein was loaded on beads (Pierce, Rockford, IL) and incubated at 4°C overnight on a rocking platform. Biotinylated and non- biotinylated proteins were separated by centrifugation, and samples were analyzed by SDS-PAGE and immunoblotting. Membranes were incubated overnight at 4°C with affinity purified rabbit anti-androgen receptor antibody (1:1000 dilution, Cell Signaling, Danvers, MA). Washes in TBST and subsequent blocking, was followed by incubation with secondary anti rabbit antibody (1:2000 dilution, Cell Signaling, Danvers, MA) for 1 h at RT. Blots were developed with the ECL detection reagent (Amersham, Freiburg, Germany). For loading controls, blots were stripped in stripping buffer (Carl Roth, Karlsruhe, Germany) at 56°C for 30 min, washed in TBST and blocked with 5% milk in TBST for 1 h at RT. After membrane incubation with affinity purified rabbit anti-β-actin antibody (1:1000 dilution, Cell Signaling, Danvers, MA) or mouse monoclonal anti-sodium potassium ATPase antibody (1:5000 dilution, Abcam, Cambridge, UK) and subsequent incubation with the respective secondary antibody, bands were detected with ECL detection reagent.

### Measurement of the G/total actin ratio by Triton X-100 fractionation

The Triton X-100-soluble G-actin-containing and total-actin-containing fractions of cells exposed to testosterone-HSA in the presence (1 h pre-treatment) or absence of 10^-7 ^M cytochalasin B (Biomol Research Laboratories, PA) were prepared as previously described [25]. A decrease in the triton-soluble (G-) over the total (T-) actin ratio is indicative of actin polymerization.

### Annexin V staining

Cells were transferred to a staining tube and washed with 4 ml PBS containing 1% BSA at 4°C. After medium removal, the cell pellet was re-suspended in 200 μl cold PBS. 5 μl Annexin V-FITC (BD Biosciences) was added and incubation was carried out for 20 min at 37°C protected from light. Then, the suspension was transferred onto a glass slide and mounted with Prolong^® ^Gold antifade reagent (Invitrogen).

### APOPercentage apoptosis assay

Caco2 cells (in RPMI 1640, supplemented with 25 mM HEPES, 2 mM L-Glutamine and 10% FBS) were cultured in 96-well plates for the APOPercentage apoptosis assay (Biocolor Ltd., Belfast, Ireland). In the presence or absence of 10^-7 ^M flutamide, cytochalasin B (Sigma) and DEVD-fmk, they were stimulated or not for 24 h in serum-containing medium with 10^-7 ^M of the following steroids: testosterone-HSA, dihydrotestosterone (DHT) and estradiol (E2). Untreated cells cultured in serum-free medium were used as positive control for apoptosis.

### Caspase-3 assay

The activity of caspase-3 was measured in whole cell lysates, pretreated or not with either 10^-7 ^M cytochalasin B, or 10^-7 ^M flutamide and then stimulated with 10^-7 ^M testosterone-HSA for the time periods indicated in the figure legends, using the Clontech ApoAlert^® ^Caspase Colorimetric Assay kit according to the manufacturers' instructions. Caspase-3 activity was determined by incubating lysates with a caspase-3 substrate (the peptide DEVD conjugated to the chromophor p-nitroaniline) for 2 h at 37°C. The absorbance of each sample was measured at 405 nm by using a 96-well colorimetric plate reader.

### In vivo experiments

Experiments were carried out on 7-week old wild-type Balb/c mice of either sex. The animals were housed under controlled environmental conditions (22-24°C, 50-70% humidity and a 12 h light/dark cycle). Throughout the study the mice had free access to standard pelleted food (C1310, Altromin, Heidenau, Germany) and tap water. All animal experiments were conducted according to the German law for the care and welfare of animals and were approved by local authorities

### Induction of colon carcinoma

Colon carcinoma was generated as described previously [26]. In a first series of experiments, 7-week old Balb/c mice (both male and female) were divided into two groups, A (n = 5) and B (n = 7). Both groups underwent carcinogenic treatment. At the age of 9 weeks animals were subjected to three cycles of alternating administration of distilled water containing 30 g/L synthetic dextran sulfate sodium (DSS; molelucar mass 5000 Da; Wako Pure Chemical Industries, Led. Japan) for 7 days followed by distilled water for subsequent 14 days after intraperitoneal pretreatment with 20 mg/kg 1, 2-dimethylhydrazine (DMH; Sigma-Aldrich Corp. St.Louis. MO. USA). Group B mice received in addition to the carcinogenic treatment 5 mg/kg testosterone-HSA subcutaneously injected three times per week throughout the study period. All mice were sacrificed at the age of 20 weeks. After death, the entire colorectum from the colorectal junction to the anal verge was examined. Fresh specimens were placed in liquid nitrogen and subsequently stored at -80°C for further analysis. Then, the colon was opened longitudinally, washed with PBS, and divided into three portions (proximal, middle and distal). After macroscopic inspection the colon was fixed in a 40% g/L formaldehyde buffer solution (pH.7.4).

### TUNEL assay

The colonic cancer tissue was cut to 8 μm frozen sections from mouse colon tumors and subsequently fixed in 4% paraformaldehyde for 30 min at room temperature. After rinsing with PBS the samples were permeabilized in a solution of 0.1% Triton X-100 in sodium citrate for 2 min. Samples were washed with PBS and incubated in the TUNEL reaction mix for 1 h at 37°C, according to the manufacturer's instructions (Roche, Germany). Nuclei were stained with DRAQ5™ (Biostatus Limited). Sections were analysed by confocal microscopy.

### Statistical analysis

Data are provided as means ± SEM, *n *represents the number of independent experiments. Data were tested for significance using unpaired student's t-test, when two-sample means were tested. Differences were considered statistically significant when *p*-values were < 0.05. All statistical analysis was performed with GraphPad InStat version 3.00 for Windows 95, GraphPad Software, San Diego California USA, http://www.graphpad.com.

## Results

### mAR expression in specimens of colon tumors and colon cancer cell lines

**Figure 1:**
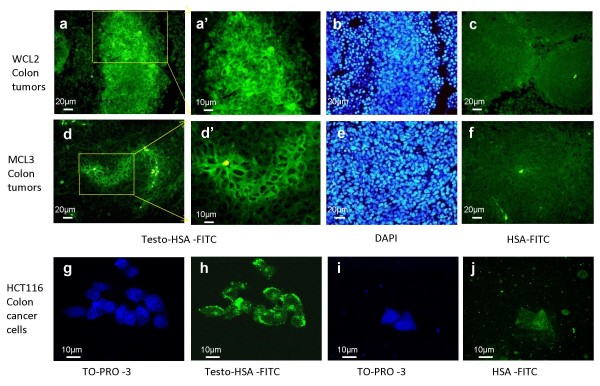
**Membrane staining of mAR in colon tumors and colon cancer cells**. Confocal laser scanning microscopic analysis of WCL2 (a-c) and MCL2 (d-f) colon tumor specimens and HCT116 cells (g-j) stained with testosterone-HSA-FITC, showing specific FITC related fluorescence at the cell membranes. Control staining with HSA-FITC showed no apparent membrane fluorescence. Visualization of nuclei was evident by DAPI or TO-PRO-3 staining. Magnification, ×100.

**Figure 2:**
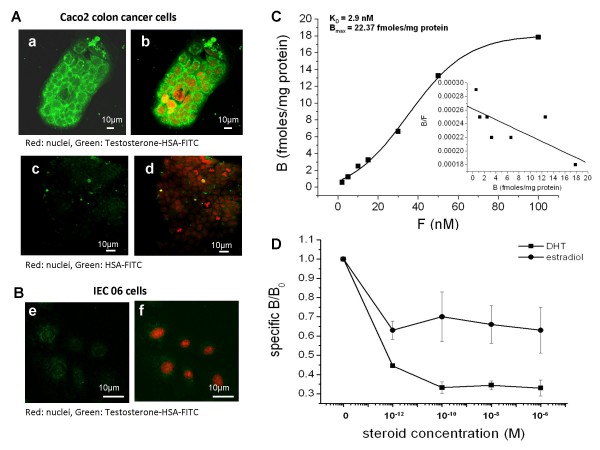
**Membrane staining and binding assays of mAR in Caco-2 cells**. A) Confocal laser scanning microscopic analysis of Caco2 cells (a-d) stained with testosterone-HSA-FITC, showing specific FITC related fluorescence at the cell membranes (a, b). No apparent membrane fluorescence was shown in control samples stained with HSA-FITC (c, d). Visualization of nuclei was evident by DRAQ5™ staining. Magnification, ×100. B) Confocal laser scanning microscopic analysis of IEC 06 cells (e-f) stained with testosterone-HSA-FITC, showing no apparent membrane fluorescence at the cell membrane. Visualization of nuclei was evident by DRAQ5™ staining. C) Saturation binding assay: Cell membranes were prepared as indicated in Materials and Methods at a final concentration of 1,2 mg/ml. They were incubated overnight at 4°C in the presence of seven concentrations of [^3^H] testosterone, which varied from 2 to 100 nM. K_D _and B_max _values for the membrane binding sites were determined from Scatchard plots (presented in inserts), based on saturation bindings. The figure represents the results of a typical experiment in triplicate. D) Displacement binding assay: Cell membranes were incubated with 5 nM of [^3^H] testosterone alone (B_o_) or in the presence of the indicated concentrations of unlabelled steroids (DHT, estradiol), ranging from 10^-12 ^to 10^-6 ^M. Nonspecific binding was assayed by introducing 5 μM DHT. The figure (means of three different experiments performed in duplicate) presents the ratio of specific binding in the presence of the indicated concentrations of DHT (B_s_) to the specific binding in the absence of DHT (B_o_), B_s_/B_o_.

While analyzing paraffin blocks generated from *in vivo *xenograft tumor tissues of various origins, we noticed significant mAR expression in colon tumors. Specifically, using testosterone-HSA-FITC fluorescent conjugates we detected specific, FITC-related fluorescence in membrane specimens of colon xenograft tumors generated from wild type HCT116 cells (WCL2) (Fig. 1a,a') or HCT116 p53-/- cells (MCL3) (Fig. 1d,d'). Conversely, no apparent staining could be identified in control tissues labeled with HSA-FITC (Fig. 1c,f). Although the apparent visualization of mAR staining in tissue preparations is restricted by technical limitations, in cultured HCT116- (Fig. 1h) or in Caco2-colon cells (Fig. 2A a,b) the membrane staining of mARs was obvious by confocal laser scanning microscopy using the fluorescent testosterone-HSA-FITC conjugate. No apparent staining was evident in HSA-FITC-labeled HCT116 or Caco2 cells (Fig. 1j, 2Ac,d). Interestingly, mAR staining could not be detected in the membrane of the non-transformed intestinal cell line IEC06 (Fig. 2B). These staining experiments and the fact that testosterone-HSA-FITC is an impermeable conjugate disclosed mAR expression preferentially in colon cancer cell lines and tumors.

### Binding studies of membrane androgen receptors in colon cancer cells

To further study the specificity of mAR we performed saturation binding of radiolabeled testosterone in membrane preparations of Caco2 cells. As shown in fig. 2C, determination of [^3^H] testosterone bound to Caco2 cells at concentrations ranging from 1 to 100 nM revealed specific saturable binding. Scatchard analysis of the results revealed high binding affinity for testosterone (K_D _2.9 nM). The calculated B_max _was 22,37 fmol/mg protein. Membrane preparations incubated with [^3^H] testosterone in the presence of varying concentrations of DHT or estradiol (10^-12 ^to 10^-6 ^M), revealed a displacement of radiolabeled testosterone by DHT (Fig. 2D), while estradiol displaced radiolabeled testosterone with a significant lower affinity, confirming the androgen selectivity of these membrane receptors.

### mAR activation triggered rapid actin and tubulin reorganization in colon cancer cells

**Figure 3:**
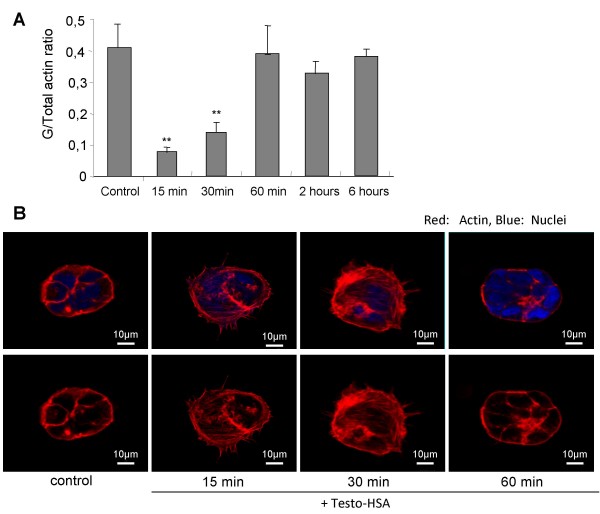
**Modulation of the dynamic equilibrium between G- and Total actin in testosterone-HSA stimulated Caco2 cells**. 24 h serum starved cells were stimulated with 10 ^-7 ^M androgen conjugate for the indicated time points. (A) Total and G- actin were measured by quantitative immunoblot analysis after Triton X-100 subcellular fractionation. Bars present the G/Total actin mean value ± SE of four independent duplicate experiments (** P < 0.01). (B) Cells were stained with rhodamine-phalloidin for filamentous actin and DRAQ5™ for nuclei. Confocal laser scanning microscopy analyzed samples. Magnification, ×100.

Cytoskeleton reorganization is a prominent early functional response of various cancer cells to steroid hormones targeting membrane binding sites [3,8,19,27]. Accordingly to analyze the functional impact of mAR in colon cancer we investigated rapid cytoskeleton modifications in Caco2 cells upon activation of mAR with testosterone-HSA for various time intervals. Cellular actin cytoskeleton dynamics were initially assessed by appropriate quantitative techniques as described in [25]. As shown in fig. 3A, quantitative immunoblot analysis of Triton X-100-insoluble cytoskeletal pellets and corresponding supernatants revealed a significant decrease of the Triton-soluble (monomeric) over total actin ratio in Caco2 cells treated with 10^-7 ^M testosterone-HSA, indicating actin polymerization. This effect was evident 15 min upon testosterone-HSA treatment and returned to nearly control levels after 1-2 h (Fig. 3A). The quantitative data were fully supported by confocal laser scanning microscopic analysis showing redistribution of microfilamentous structures and formation of stress fibers and filopodia in testosterone-HSA treated cells (Fig. 3B). We further analyzed tubulin cytoskeleton reorganization by confocal laser scanning microscopy. A clear redistribution of the microtubular network became evident in cells treated with 10^-7 ^M testosterone-HSA for 15 to 60 min (Additional File 1).

### mAR activation induced profound and specific pro-apoptotic responses in colon cancer cells

**Figure 4:**
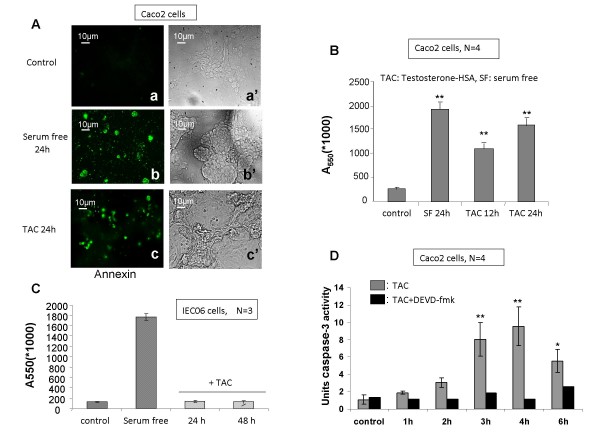
**Pro-apoptotic effects of testosterone-HSA in Caco2 cells**. Caco2 cells stimulated with 10^-7 ^M androgen conjugate for the indicated time periods. (A) Cells were stained with Annexin V and visualized by confocal laser scanning microscopy. Magnification, ×400. (B) Quantitative APO-Percentage apoptosis assay of testosterone-HSA stimulated Caco2 cells according to the manufacturer's instructions. Bars present the mean OD measured at 550 nm (** P < 0.01, N = 4). (C) Non-transformed intestinal IEC 06 cells were exposed to 10^-7 ^M testosterone-HSA for 24 and 48 hours. No apoptoric response was evident by the APOPercentage apoptosis assay. Cells serum starved for 24 hours served as positive control for apoptosis. Bars present the mean OD measured at 550 nm, N = 3. (D) Caspase-3 activity was measured at 405 nm in lysates derived from testosterone-HSA treated cells in the presence, or absence of caspase-3 inhibitor DEVD-fmk for the indicated time periods and then incubated with the caspase-3 substrate DEVD conjugated to the chromophore pNA according to the manufacturer's instructions (** P < 0.01, * P < 0.05, N = 4).

**Figure 5:**
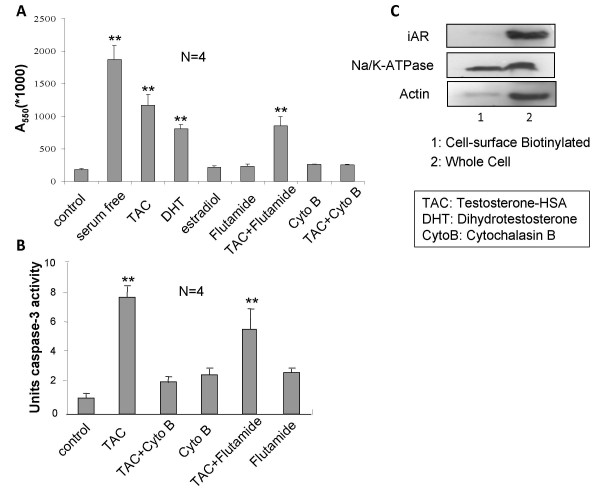
**Pro-apoptotic effects of testosterone-HSA, DHT and estradiol in the absence or presence of inhibitors in Caco2 cells**. (A) Quantitative APOPercentage apoptosis assay of testosterone-HSA, DHT or estradiol stimulated Caco2 cells and in the presence/absence of cytochalasin B (Cyto B) or flutamide. Cells were exposed to 10^-7 ^M testosterone-HSA, DHT or estradiol for 24 hours and pro-apoptoric responses were assessed by the APOPercentage apoptosis assay. Equally, cells pre-treated or not with 10^-7 ^M Cyto B or flutamide, were exposed to testosterone-HSA for 24 hours. Cells serum starved for comparable periods of time served as a positive control for apoptosis. Bars present the mean OD measured at 550 nm (** P < 0.01, N = 4). (B) Cells were pre-treated or not with Cyto B or flutamide for 1 h and then exposed or not to 10^-7 ^M testosterone-HSA for 4 h, lysed and incubated with the caspase-3 substrate DEVD conjugated to the chromophore pNA according to the manufacturer's instructions. Caspase-3 activity was measured at 405 nm (** P < 0.01, N = 4). (C) Immunoblot analysis for iAR expression in membrane preparations. Membranes were isolated as described in Materials and Methods and subjected to immunoblot analysis using a specific iAR antibody, and subsequently a Na^+^/K^+ ^ATPase or actin antibody as a positive/negative control respectively.

**Figure 6:**
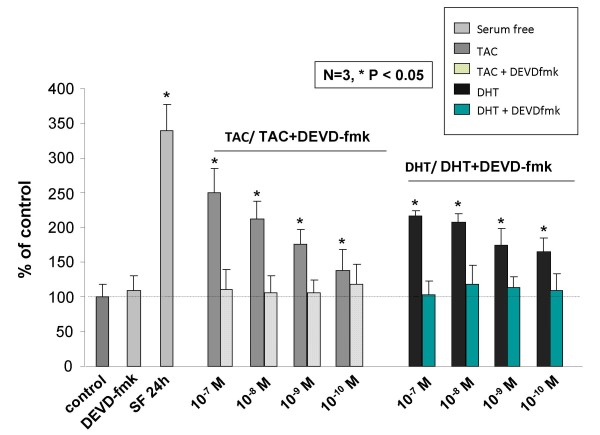
**Dose-dependent analysis of testosterone-HSA and DHT induced apoptosis in Caco2 cells in the absence or presence of caspase-3 inhibitor**. Quantitative APOPercentage apoptosis assay of testosterone-HSA and DHT stimulated Caco2 cells. Cells were exposed to 10^-7 ^M to 10^-10^M testosterone-HSA and DHT respectively in the presence or absence of the caspase-3 inhibitor DEVD-fmk for 24 hours. The pro-apoptoric response was assessed by the APOPercentage apoptosis assay. Cells serum starved for comparable period of time served as a positive control for apoptosis. Bars indicate the mean OD measured at 550 nm ± SE of three independent experiments performed in triplicates, normalized versus the untreated control (serum supplemented cells) and presented as percentage (%) of the untreated serum supplemented control cells taken as 100 (*P < 0.05, N = 3).

To further analyze the functional status of mAR activation in colon cancer cells, we assessed mAR-dependent pro-apoptotic responses in Caco2 cells, as previously reported in prostate cancer cells [8,9]. Annexin V fluorescence staining of Caco2 cells treated with 10^-7 ^M testosterone-HSA for 24 h revealed clear evidence for induction of apoptosis similar to the one observed in serum-starved cells (Fig. 4A, compare b and c). The induction of apoptosis in Caco2 cells was further evident from the quantitative APOPercentage apoptosis assay, which showed a clear testosterone-HSA-stimulated apoptotic response 12 and 24 h post-treatment (Fig. 4B). Similar results were also obtained in HCT116 cells (data not shown), while mAR deficient (Fig 2B) non-transformed IEC06 intestinal cells did not responded to testosterone-HSA treatment, as indicated by the APOPercentage apoptosis assay (Fig. 4C). In line with these findings, testosterone-HSA induced time-dependent activation of caspase-3 (Fig. 4D), indicating the participation of caspases as executors in mAR-dependent cell death. These effects were attributed to mAR activation and were independent of classical intracellular androgen receptors, since both the apoptotic response (Fig. 5A) and the caspase-3 activation (Fig. 5B) were not inhibited in the presence of the anti-androgen flutamide. In line with this, membrane-bound iAR could not be detected in isolated membrane preparations of Caco2 cells by using specific iAR antibodies (Fig. 5C). In contrast, these membrane preparations were positive for the expression of Na/K ATPase, a protein implicated in cellular ion homeostasis used as a positive membrane control in this experiment. To establish the functional role of actin reorganization in regulating the pro-apoptotic responses induced by mAR, as previously reported for various cell systems [8,28,29], we assessed mAR-dependent apoptosis and caspase-3 activation in the presence of anti-actin drugs. As shown in Fig. 5A, B, in Caco2 cells pre-treated with cytochalasin B, at a concentration (10^-7^M) which blocks actin redistribution without exerting toxic effects [30], the mAR-induced apoptotic response (Fig. 5A) and caspase-3 activation (Fig. 5B) were abolished. These results indicate that actin redistribution is a mandatory step for the apoptotic response of mAR-stimulated colon cancer cells. We further evaluated the steroid-hormone specificity of the mAR-induced apoptotic responses by using non-conjugated testosterone and estradiol derivatives. As shown in fig. 5A, free estradiol could not generate any apoptotic response, while free DHT clearly showed activity. Finally, considering the estimated K_D _of 2.9 nM for mAR (Fig. 2B, C), we further performed titration experiments using a wide range of testosterone-HSA and free DHT concentrations (10^-7 ^M to 10^-10 ^M) in the presence or absence of caspase inhibitor respectively. These experiments indicate that even in the nM range the testosterone conjugate and DHT have very similar pro-apoptotic effects (Fig. 6). These effects were abolished in the presence of the caspase inhibitor (Fig. 6).

### mAR activation by testosterone-HSA was followed by extensive reduction of tumor incidence *in vivo*

**Figure 7:**
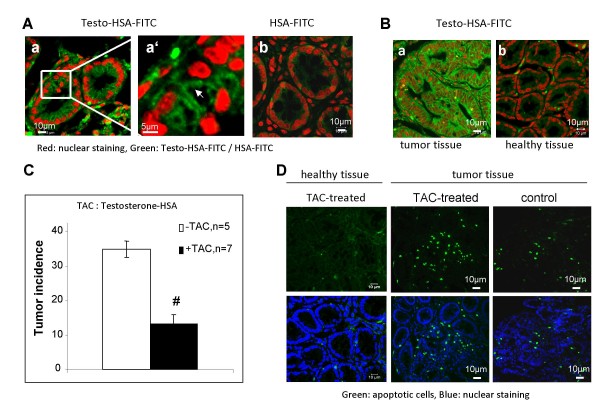
***In vivo *testosterone-HSA effects on tumor incidence in BALB/c mice**. A) Confocal laser scanning microscopic analysis of BALB/c colon tumor frozen sections stained with testosterone-HSA-FITC (a, a'), showing specific FITC related fluorescence at the cell membranes. No apparent membrane fluorescence was shown in control samples stained with HSA-FITC (b). Visualization of nuclei was evident by DRAQ5™ staining. Magnification, ×100. (B) Confocal laser scanning microscopic analysis of BALB/c colon tumor and healthy frozen sections stained with testosterone-HSA-FITC, showing specific FITC related fluorescence at the cell membranes of tumor sections (a). Very low membrane fluorescence was shown in healthy colon sections stained with testosterone-HSA-FITC (b). Visualization of nuclei was evident by DRAQ5™ staining. Magnification, ×100. (C) Arithmetic means ± SEM of colonic tumor incidence in BALB/c mice. Following treatment with the carcinogenic drug 1, 2-dimethylhydrazine followed by dextrane sodium sulphate, one group (7 animals) was treated subcutaneously (3 times/week for 12 weeks) with 5 mg/kg testosterone-HAS (black bar), whereas the other group (5 animals) remained untreated (white bar). # indicates significant difference between both groups (# P < 0.01). (D) After treatment, the colonic cancer and healthy tissue was cut to 8 μm frozen sections and fragmented DNA was assessed by TUNEL assay according to the manufacturer's instructions. Confocal laser scanning microscopy analyzed samples. Magnification, ×100.

The findings provided so far established that mAR activation results in colon cancer cell regression *in vitro*. Thus, we aimed to further evaluate the *in vivo *effects of albumin-conjugated androgens in colon cancer animal models. To this end, we first estimated the expression of mAR in colon tumors generated in Balb/c mice. As shown in fig. 7A and fig. 7B, using testosterone-HSA-FITC we detected specific, FITC-related fluorescence in membrane specimens of Balb/c mice colon tumors (Fig. 7Aa, a'), while no apparent staining could be identified in tissues labeled with HSA-FITC (Fig. 7Ab). Interestingly, mAR staining was very low in healthy colon tissue specimens of Balb/c mice (Fig. 7B) further supporting earlier findings pointing to cancer tissue specificity of mAR [31]. Having a clear indication for mAR-expression, we assessed the 12-week tumor incidence of colon tumors generated in Balb/c mice by chemical carcinogenesis (see Materials and Methods) in the presence or absence of continuous testosterone-HSA treatment. The animals used for these studies were divided in two groups comprising of 5 and 7 animals respectively. One group (7 animals) was treated subcutaneously (3 times/week for 12 weeks) with 5 mg/kg testosterone-HSA, whereas the other group (5 animals) remained untreated. The results (Fig. 7C) show that testosterone-HSA treatment produced a clear and significant reduction of tumor incidence by 65%. The histological analysis of tumors by TUNEL assay confirmed that apoptotic cells were present in significant numbers predominantly in the tumors of animals treated with testosterone-HSA (Fig. 7D, middle panels), while they were significantly less either in the non-treated animals (Fig. 7D, right panels), or in healthy tissues of treated animals (Fig. 7D, left panels). These results collectively show that mAR is a functional and specific target that may be used for the selective elimination of colon cancer cells *in vivo*.

## Discussion

In the present work we provide strong experimental evidence that membrane androgen receptors are expressed in colon tumors. Using tissue specimens derived from colon tumors and established colon tumor cell lines we showed that colon cancer cells predominantly express functional mAR, while mAR expression is undetectable in healthy mouse colon tissues or non-transformed intestinal cells. Moreover, membrane-impermeable testosterone albumin conjugates induced a) profound and rapid actin and tubulin reorganization, and b) considerable apoptosis via activation of the pro-apoptotic executor caspase-3. The observed mAR-activated effects were specific for testosterone and testosterone conjugates, since other steroid hormones such as estradiol did not exhibit any pro-apoptotic activity.

Our binding studies, including radioligand binding experiments, Scatchard analysis and displacement assays, attributed receptor properties to the identified testosterone membrane binding sites. Moreover, they showed high affinity and specificity for testosterone, as indicated by the displacement of radiolabeled testosterone by DHT but not by estradiol. The calculated K_D _(2.9 nM) is very close to that reported for LNCaP and DU145 prostate cancer cells [3,7]. Others also obtained similar results in different cell systems [32]. These data collectively indicate that these membrane-binding sites may potentially represent a specific membrane receptor for androgens. Hitherto the exact molecular identity of mAR remained elusive. According to the experimental evidence provided so far, mAR may represent either (I) a pool of iAR targeted to the plasma membrane and/or associated membrane structures (e.g. lipid rafts or caveolae) mediating rapid androgen effects in the absence of transcriptional activity [33] or (II) an unknown G-protein-coupled receptor (GPCR) (or a receptor associated with a GPCR) triggering a variety of iAR-independent signaling cascades [17,34,35]. Our results, showing that iAR a) could not be detected in membrane preparations of colon cancer cells (Fig. 5C) and b) that testosterone-HSA effects were manifested even in the presence of the anti-androgen flutamide (Fig 5A, B), imply that the molecular identity of mAR is probably not identical with iAR, targeted to the plasma membrane. They are in line with previous reports for LNCaP and DU-145 prostate cancer cells [7,8]. Although these findings argue against the hypothesis that iAR is expressed in the plasma membrane, only the identification and/or molecular cloning of this new receptor can define his molecular identity.

The results from this and other studies indicated that membrane androgen receptors are predominantly expressed in tumor cells ([31] and Fig. 2B, 7B). In addition, activation of these receptors triggers pro-apoptotic responses. One possible rationalization for the expression of those receptors is that tumor cells may compensate mAR-dependent apoptosis by over-expressing anti-apoptotic proteins or other compensatory mechanisms that collectively protect against mAR-dependent apoptosis. Previous reports support this assumption: Indeed, iAR-deficient DU145 human prostate cancer cells were shown to overexpress the pro-survival PI-3K/Akt pathway, which was down-regulated following long-term mAR activation [9]. In addition, the FAK/PI3K pathway was constitutively activated in DU145 cells and mAR activation was unable to further alter the short-term phosphorylation levels of those kinases [8], while long term activation induced significant de-phosphorylation [9].

The connection between actin cytoskeleton components and androgen signaling has attracted specific interest in recent years (for a review see [36]). Actin dynamics seem to be crucial for apoptotic responses [28,29]. The findings in our present work further underscored the key role of actin cytoskeleton rearrangements in regulating apoptosis. Indeed, it was clearly shown that actin (and tubulin) reorganization represent major early events following mAR activation by testosterone-HSA. Moreover, early blockade of actin rearrangement by depolymerizing drugs e.g. cytochalasin B, virtually abrogated the pro-apoptotic responses (Fig. 5A, B). The involvement of the early actin rearrangement in mediating the late apoptotic responses was addressed in earlier studies in prostate cancer cells. In these studies it was shown that inhibition of either up-stream or down-stream signals regulating early actin polymerization blocked the late activation of NF-κB and FasL signaling [9]. Although the pro-apoptotic signaling was not addressed in the present study we hypothesize that the actin reorganization is an early functional step in the pro-apoptotic response. These findings, which are in close agreement with similar results reported recently in prostate cancer cells treated with testosterone albumin conjugates [8,9], further emphasize the functional cross-talk between cytoskeleton rearrangements and regulation of apoptosis [28,29].

Recent studies using mouse xenografts have shown that a testosterone-albumin conjugate (testosterone-BSA) induced potent apoptotic regression of prostate tumors *in vivo *[7]. In addition, testosterone-BSA was also reported to potentiate the paclitaxel-mediated cytotoxicity both *in vitro *and *in vivo *[19]. Based on these reports, on the expression patterns indicating predominant mAR manifestation in cancer cells (Fig. 1, 2A, 2B and 7A, 7B) and on the functional analysis of those receptors in colon cancer specimens and cell lines (Fig. 4, 5, 6), we evaluated their potential biological role as drug targets in colon tumors *in vivo*. Interestingly, the chemically-induced colon tumors were reduced by 65% in the testosterone-HSA-treated animals. Most probably this effect was due to the apoptotic regression of tumor cells as indicated by the TUNEL assay (Fig. 7D, middle panel). These results point out clearly that activation of mAR by testosterone-HSA significantly affects the incidence of colon tumors *in vivo*. Interestingly, mAR is strongly expressed in tissues derived from p53-deficient xenograft tumors (Fig. 1d, d'). Since p53 is a frequently inactivated gene in tumors, it is interesting to hypothesize that mAR activation may result in eradication of p53 tumors *in vivo*. In addition, the detailed analysis of mAR expression in normal and cancer colon tissues isolated from mice revealed clearly mAR over-expression in tumor tissues, while in healthy specimens and non transformed intestinal IEC06 cells mAR expression was undetectable (Fig. 2B, 7B). These findings support the notion that most probably normal cells will not respond to testosterone-HSA treatment, a conclusion supported by the TUNEL assay (Fig. 7D, left panels), which indicated very low apoptotic response of normal tissues to testosterone-HSA treatment, as well as from the failure of any pro-apoptotic response in testosterone-HSA treated IEC06 cells (Fig. 4C). Despite the fact that additional experiments are required for the detailed evaluation of mAR-dependent biological effects in colon cancer, our findings fully enforce the potential significance of the recently postulated notion [17] that mAR may represent a novel and specific tumor target.

In conclusion, the results presented here add a clear and significant piece of evidence to the potential anti-tumorigenic role of membrane androgen receptors. They indicate that a) functional mAR are expressed not only in hormone-dependent tumors but also in colon tumors, b) their activation through steroid albumin conjugates induces potent pro-apoptotic responses regulated by cytoskeletal rearrangements, and c) these receptors may represent specific targets for the development of novel drugs, since their activation drastically regresses tumor growth and tumor incidence *in vivo*. Additional experiments are now required for the identification of the molecular identity of these receptors.

## Competing interests

The authors declare that they have no competing interests.

## Authors' contributions

SG and NP carried out the mAR staining in colon tumors and cell lines, the analysis of actin and tubulin reorganization and the pro-apoptotic responses. EMG carried out the binding studies. ON performed the animal experiments. KD prepared the mouse xenografts. SKB carried out the flutamide and cytochalasin B control experiments. MF participated in the design of the study and performed the statistical analysis. KA participated in the design of the study and drafting of the manuscript. FL participated in the coordination of the study and evaluation of the results. CS conceived of the study, participated in the design coordination and drafting of the manuscript. All authors read and approved the final manuscript.

## Supplementary Material

Additional file 1 **Rapid tubulin reorganization in testosterone-HSA stimulated Caco2 cells**. Caco2 cells treated or not with 10^-7 ^M testosterone-HSA for different time points were cultured in coverslips, fixed and stained with rabbit anti-α-tubulin. Anti-rabbit-FITC was used as secondary antibody and DRAQ5™ for nuclei staining. Confocal laser scanning microscopy analyzed samples. Magnification, ×100. Click here for file
