# Supplementary figures and images for: Functional membrane androgen receptors in colon tumors trigger pro-apoptotic responses in vitro and reduce drastically tumor incidence in vivo
Source: Mol Cancer. 2009 Dec 1;8:114. doi: 10.1186/1476-4598-8-114 (PMC2794856; doi:10.1186/1476-4598-8-114)

## Slide 1
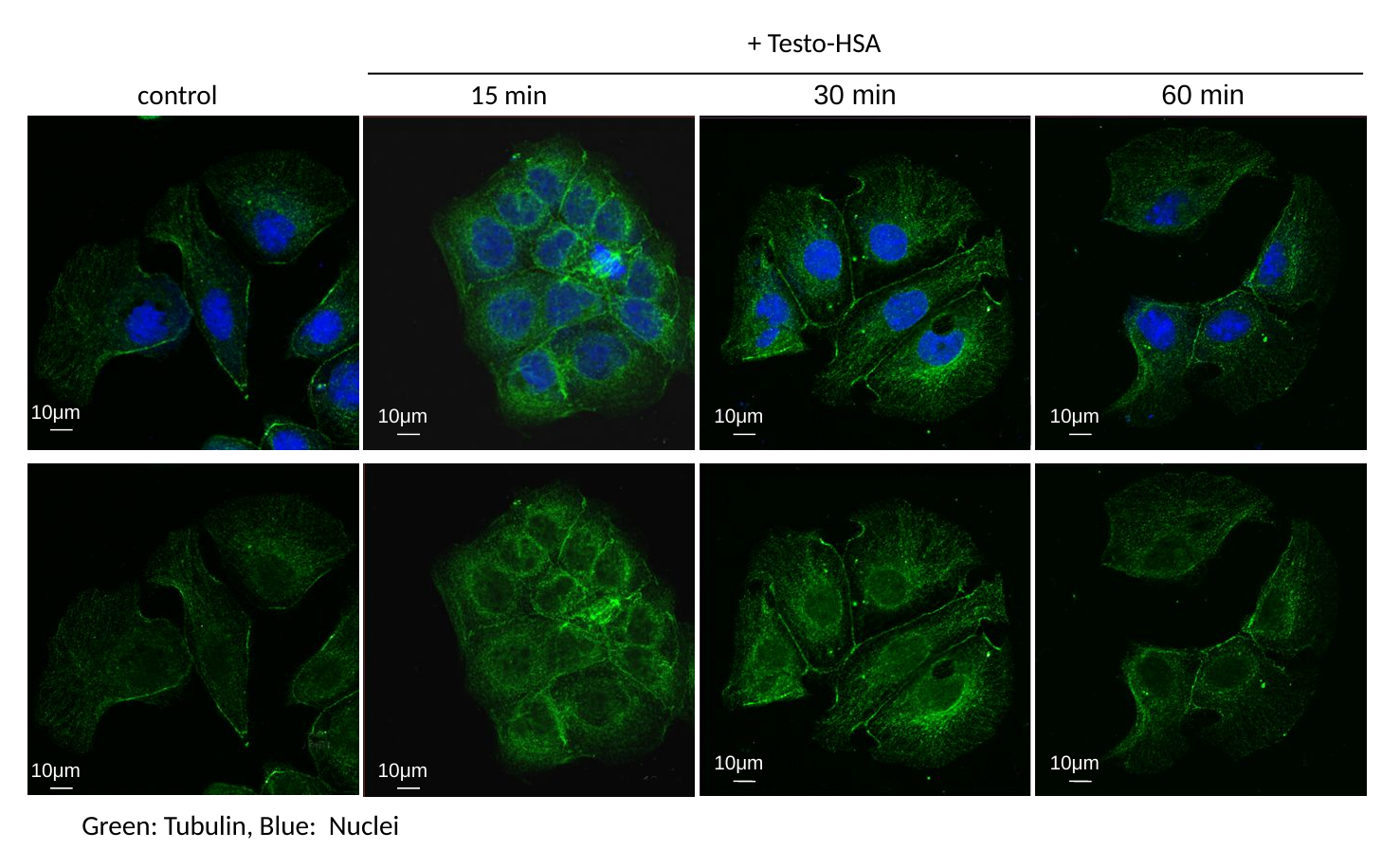

+ Testo-HSA
control
15 min
30 min
60 min
10μm
10μm
10μm
10μm
10μm
10μm
10μm
10μm
Green: Tubulin, Blue: Nuclei

Supplement: Additional file 1 — Rapid tubulin reorganization in testosterone-HSA stimulated Caco2 cells. Caco2 cells treated or not with 10-7 M testosterone-HSA for different time points were cultured in coverslips, fixed and stained with rabbit anti-α-tubulin. Anti-rabbit-FITC was used as secondary antibody and DRAQ5™ for nuclei staining. Confocal laser scanning microscopy analyzed samples. Magnification, ×100. [file 1476-4598-8-114-S1.PPT]
